# COVID-19 and the Otolaryngology Match: An Increase in Applicants Remaining Close to Home

**DOI:** 10.7759/cureus.23650

**Published:** 2022-03-30

**Authors:** Cees T Whisonant, Shawhin R Shahriari, Casey D McDonald, Addi N Moya, Amanda Ederle, Gregory Borah

**Affiliations:** 1 Division of Plastic, Reconstructive, Hand and Burn Surgery, Department of Surgery, University of New Mexico School of Medicine, Albuquerque, USA; 2 Department of Internal Medicine, Baptist Health, Little Rock, USA

**Keywords:** otolaryngology residency, covid-19, the match, residency, otolaryngology

## Abstract

Introduction: Otolaryngology residency remains one of the most competitive surgical subspecialties to apply for with a 63% match rate in 2021. This is a difficult and stressful process for applicants in any given year, and it was even further complicated by restrictions mandated by coronavirus disease 2019 (COVID-19) protections. Analyzing geographical trends in successfully matched applicants provides prospective applicants and programs with helpful information about how previous trends were affected by the pandemic as we look toward future match cycles.

Methods: The medical schools of 1,587 successfully matched applicants from 2017-2021 were identified and compared to the 116 otolaryngology residency programs. Successful applicants’ medical school state and region were then compared to the location of their matched residency program state and region. From this, we evaluated the number of applicants matching at the residency program affiliated with their medical school or at a residency program within the same state or region as their home medical school.

Results: A significant increase in the percentage of applicants matching at their home program and within their home state (p < 0.001) occurred in 2021 when compared to previous years. Applicants matching within their home region was not found to increase significantly (p = 0.43) in 2021 compared to previously. The regions with the greatest increase in the percentage of applicants matching to their home programs were the Northeast and Midwest (12% increase), while the Midwest had the largest increase in percentage of applicants matching within their home state (15%).

Conclusion: The COVID-19 pandemic significantly affected the otolaryngology match in 2021 with applicants and programs alike choosing to stay closer to home with their residency match selections. Overall, regional location remains a major determinant of future residency location for otolaryngology applicants, and this did not change significantly during 2021, but applicants matched more frequently at their home medical school program. It is anticipated that the match process will be similar in 2022 given the ongoing pandemic, so the importance of home program and region will likely be emphasized again.

## Introduction

Otolaryngology is one of the most competitive surgical residencies to successfully match into. Over 550 medical students applied to match at one of 350 otolaryngology residency positions in 2021 [1]. Between 2015-2020, the number of students applying increased by 9.3% each year, however, there was only a 3.6% increase annually in training positions [2]. Medical students may apply to more programs with the thought that this will increase their chances of matching into their preferred specialty. Acceptance rates, however, have not been found to correlate with the mean number of applications [3]. Subsequent studies have attempted to identify what makes a competitive applicant more likely to match, including personal connections to a program or region and the effects that the COVID-19 pandemic had on the otolaryngology residency match [4-6]. 

The COVID-19 pandemic took away much of the personal aspect of the application process for both applicants and residency programs in 2021, specifically through reduced or absent away rotations [7]. This was further compounded by the requirement for virtual residency interviews which did not allow for applicants to visit in person and get to experience the residency locations and programs first-hand [8]. The pandemic has continued to impact medical education for the class of 2022 with away rotations being limited to one per student, and the release of the recommendation for another round of virtual interviews in 2022 by the Coalition for Physician Accountability [9]. With this in mind, it is important that applicants and otolaryngology programs have a general idea of how a similar situation previously affected the Match in 2021. Although it remains unclear how the Match will proceed in the coming years, some aspects of the application process that have emerged as the result of the pandemic, such as virtual interviews, are likely to continue. This study aims to identify recent trends in the otolaryngology Match process by evaluating applicants matching at their home program (HP) i.e., the residency program affiliated with their medical school, within their home state (HS), and within their home region (HR) during the 2017-2021 Match cycles. This study also aimed to determine how these variables related to the Match changed during the 2021 Match cycle when compared to previous years.

## Materials and methods

A five-year study period, that parallels the length of a typical otolaryngology residency, was selected encompassing the most recent years (2017-2021) of the otolaryngology Match. Otolaryngology residency programs accredited by the Accreditation Council for Graduate Medical Education, and the number of positions offered by each respective program, was obtained through the National Resident Matching Program website [10]. Otolaryngology residency training program’s websites and their respective social media accounts were accessed to determine the medical school previously attended by residents at these training institutions. The residents’ medical school location was further characterized by state and region according to the United States Census. Current residents were then categorized as matching at the same institution as where they attended medical school (home program), within the same state as where they attended medical school (home state), within the same geographic region as where they attended medical school (home region), or none of the above. The collected data was analyzed to determine the number and percentages of residents matching within their home region or home state as well as at the residency program associated with their medical school over the five-year study period. Chi-square analysis of the data was performed to determine if any trends with regard to geographic locations of medical schools attended and residency programs of otolaryngology residents existed. A p-value of less than 0.05 considered to be statistically significant.

## Results

**Figure 1:**
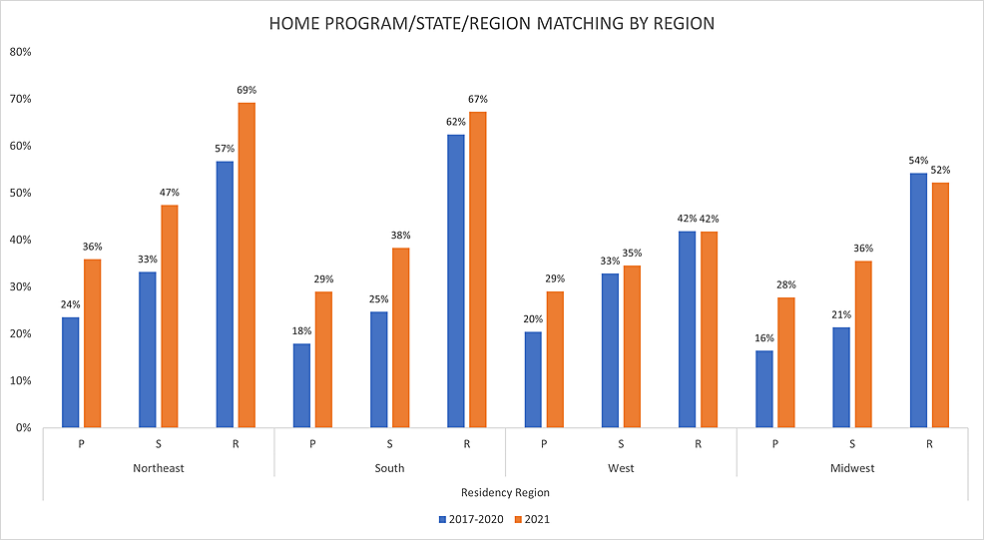
Residency Region Effect on Percent of Home Program/State/Region Matching P - program, S - state, R - region

1587 successfully matched otolaryngology residency applicants’ data were collected and analyzed for the years 2017 to 2021, accounting for 97% of all matched applicants during the study period. Table 1 shows the total number of matched applicants per year as well as the total number matching at their home program, within their home state, or within their home region. Figure 1 demonstrates home program, state, and region matching by residency program region.

**Table 1:** Year Effect on Home Program/State/Region Matching *indicates statistical significance

			Home Program		Home State		Home Region	
Year	N		n	%	n	%	n	%
2017	301		54	18%	77	26%	160	53%
2018	309		49	16%	75	24%	163	53%
2019	329		69	21%	84	26%	180	55%
2020	349		70	20%	105	30%	190	54%
2021	349		100	29%	129	37%	196	56%
2017-2021			p-value < 0.001*		p-value = 0.001*		p-value = 0.91	
2017-2020			p-value = 0.35		p-value = 0.34		p-value = 0.95	
2017-2020 vs 2021			p-value < 0.001*		p-value < 0.001*		p-value = 0.43	

Home program matching

A significant increase (p < 0.001) in the percentage of applicants matching at the program affiliated with their medical school occurred between 2017 to 2021 (Table 1). When the data were further analyzed, no significant increase in home matching occurred from 2017 to 2020 (p = 0.35), however, a statistically significant increase (p < 0.001) occurred when the data from 2017 to 2020 were compared to the data from 2021. Figure 1 shows that the greatest increase in HP matching occurred in the Northeast and Midwest (12% increase), although HP matching increased in both the West (9% increase) and the South (11% increase) as well.

Home state matching

HS matching was found to increase significantly (p = 0.001) from 2017-2021. Once again, when the data were further analyzed no significant increase occurred between 2017-2020 (p = 0.34). When the data from 2017-2020 was analyzed against the data from 2021, there was an increase in the percentage of HS matches from an average of 26% between 2017-2020 to 37% in 2021 (p < 0.001). In 2021, HS matching increased by 15% in the Midwest when compared to previous years, the greatest increase of any region. HS matching increased in all three other regions as well in 2021 compared to previous years (Northeast 14%, South 13%, West 2%).

Regional matching

HR matching was not found to change significantly from 2017-2021 (p = 0.91), nor when comparing 2017-2020 versus 2021 (p = 0.43). While the Northeast and South saw an increased percentage of 12% and 5% of HR matches, respectively, HR matching in the West remained unchanged and decreased 2% in the Midwest.

## Discussion

COVID-19 has dramatically changed the way residency programs and applicants interview and interact with each other. Current recommendations across the United States call for virtual away rotations and place a limit on the amount of rotations students can complete at training institutions not affiliated with their medical school. This inherently decreased the number of programs a single applicant was exposed to while simultaneously decreasing the number of prospective applicants a program was exposed to. Due to these restrictions, programs may have had a limited ability to assess applicants, and vice versa [11]. Considering the current trend of increasing competitiveness of applicants year after year, successfully matching the best-fitting individual to their best program has become increasingly challenging [4-6].

Lenze et al. utilized data from the Texas STAR Survey for otolaryngology residents and revealed that from 2018 to 2020, 79% of matched applicants reported matching at a program having a connection in either away rotation, personal geographic connection, or medical school in the same geographic area [4]. The results of our study were found to be in agreement with this previous study as the percentage of HP matches was high from 2017-2021. With this in mind, it is not surprising that the pandemic seems to have favored HP matches among applicants and programs with a statistically significant increase seen in 2021.

A 2015 study of 810 otolaryngology residents showed that 21% of residents matched at the program affiliated with their medical school. The Midwest was found to have the highest proportion of HP matches at over 25% versus the West where HP matches accounted for 12.5% of current residents [5]. In contrast, in our study between 2016-2020 as well as in 2021, the Northeast was found to have the highest percentage of HP matches of 24% and 38%, respectively (Figure 1). When comparing HP matches in 2021 to previous years, as shown in Table 1, a statistically significant increase occurred. The Northeast and Midwest regions had the largest increase of any region (12% increase) in HP matches. Regardless of region, an overall increase was observed as well during the study period from 19% HP matched applicants on average between 2016-2020 to 29% HP matched applicants in 2021. Two recent studies also identified an increase in applicants matching at their respective home programs [6,12]. Additionally, this was also similar to what was seen in the integrated plastic surgery match, another competitive surgical specialty, in 2021 where there was a 9% increase in HP matches [13]. Similar trends are also seen outside the surgical residency programs, with a 9.1% increase in HP matches seen in dermatology in 2021 [14]. A recent survey identified that 25% of medical student respondents did not have an otolaryngology home program [15]. Future studies investigating match rates of applicants with and without a home program, within the context of COVID-19 and its constraints, would be beneficial to further characterize the overall impact of COVID-19.

As shown in Table 1, HS matches also saw a significant increase of more than 10% in 2021. States most likely to match HS applicants are listed in Table 2. Of note, programs in New York saw a 19% increase in HS matches, the greatest increase of any state (56% in 2021 from 37% previously). Interestingly, other states in Table 2 were found to remain similar or even decrease in percentage of HS matches. 

**Table 2:** States Most Likely to Match Same State Graduates

	2017-2020			2021		
State	Positions	Same State	%	Positions	Same State	%
California	130	59	45%	35	13	37%
New York	115	42	37%	34	19	56%
Pennsylvania	81	26	32%	25	8	32%
Texas	74	28	38%	19	8	42%

It was previously reported that 58% of residents attended a program within the same region as their medical school. The highest percentage of HR matches was found in the South at 68% and the West had the lowest proportion at 31% [5]. Our study showed similar results. HR matches did not change significantly over the study period, with an average HR match rate of 54% in 2017-2020 and 56% in 2021. It is possible that this is simply due to the volume of applicants matching within their own region prior to the pandemic, however, it is interesting that despite an increase in HP and HS matches, HR matches did not increase proportionally. This data may also be skewed by graduates staying close to home but matching at programs in neighboring states that happen to be in a geographically different region.

This study did not account for competitive factors, research years, back-filled spots, or away rotations completed by applicants at residency programs. In addition, we did not include students who did not successfully match into our analysis, as our goal was to analyze geographic trends in successful matriculants. The use of residency websites and social media accounts for data collection was also a limitation. The lack of opportunity in the 2021 cycle to attend an away rotation limited programs and could have shifted their focus toward home applicants. This could explain our results.

## Conclusions

Otolaryngology residency applicants matriculating to residency programs affiliated with their medical school as well as training programs within the same state or region increased during the COVID-19 affected 2021 match cycle as compared to previous years. Limitations on in-person evaluations during away rotations likely made exposure to students’ home and nearby residency programs all that much more important. With away rotations being limited in the 2021-2022 application cycle, and most programs likely continuing to interview applicants virtually, it is important that applicants be aware of the importance of their exposure to residency programs. We hope applicants can use this study to prioritize the programs that are most likely to take them seriously as prospective residents and provide insight to both applicants and programs about current trends in the otolaryngology match. By realizing these trends, both applicants and programs can learn and adjust for future match cycles.
